# Identifying Active *Salmonella* Infections in Swine Nurseries Using Serology and Bacterial Culture and Evaluating Associated Risk Factors

**DOI:** 10.3390/ani10091517

**Published:** 2020-08-27

**Authors:** Saranya Nair, Abdolvahab Farzan, Zvonimir Poljak, Robert Friendship

**Affiliations:** Department of Population Medicine, University of Guelph, Guelph, ON N1G 2W1, Canada; nairs@uoguelph.ca (S.N.); afarzan@uoguelph.ca (A.F.); zpoljak@uoguelph.ca (Z.P.)

**Keywords:** swine, nursery pigs, epidemiology, *Salmonella*, risk factors

## Abstract

**Simple Summary:**

The presence of *Salmonella* on farms is a concern to the swine industry. Much of the focus of on-farm surveillance has been directed to the finishing stage because of food safety issues, but it is important to study *Salmonella* transmission during the nursery stage in order to develop control strategies. In this study, 50 cohorts of weaned pigs were monitored for *Salmonella* using blood samples taken at weaning and again near the end of the nursery stage and tested for antibodies. At the time of the second blood sampling, rectal swabs were obtained from the same pigs and cultured for *Salmonella*. A questionnaire regarding housing and management was also completed and used to evaluate risk factors for herds with active infection. If one pig out of the 20 tested in a cohort was found to be positive either based on the growth of *Salmonella* on culture or a rising antibody titre, then it was assumed that *Salmonella* was spreading among the pigs in that cohort. Active spread of *Salmonella* occurred in 80% of the nursery cohorts. Unfortunately, no risk factors were identified to explain the difference between positive and negative nurseries, including whether or not the farm used antibiotics.

**Abstract:**

The objectives of this study were: to identify nursery cohorts with an active *Salmonella* infection using combined serological and bacteriological methods, and to try to identify risk factors associated with swine nurseries with active *Salmonella* spread. Twenty pigs from each of 50 cohorts of weaned pigs from 44 different nursery barns were sampled about the time of weaning and near the end of the nursery stage. Information regarding farm management and biosecurity practices were collected using a questionnaire. Blood samples were obtained at both visits, while rectal swabs were collected at the second visit. An enzyme-linked immunosorbent assay (ELISA) was used to test sera for *Salmonella* antibodies and rectal samples were cultured for *Salmonella*. A nursery cohort was identified as having an active *Salmonella* infection if *Salmonella* was cultured from one or more of the 20 pigs or if serological evidence suggested exposure to *Salmonella*. The association between farm-level management covariates and active *Salmonella* infection was assessed in 46 cohorts using a logistic regression model. Nine of 46 (20%) cohorts produced *Salmonella*-free pigs. The remaining 37 (80%) cohorts were classified as having an active infection. Examination of risk factors failed to identify how negative and positive nurseries differed.

## 1. Introduction

*Salmonella* infection is an important concern for the pig industry, primarily from a public health standpoint but also as a cause of economically important clinical disease in pigs [1,2,3]. The epidemiology of *Salmonella* in pork production has been widely studied [1,3,4,5,6,7,8,9,10,11,12,13,14]. However, the emphasis has generally been placed on understanding the prevalence at the finishing stage, because of food safety concerns [9,10,11,14]. However, as with many diseases, stopping the spread of *Salmonella* in the nursery might be an important strategy in reducing the prevalence of *Salmonella* in later stages of production. Identifying the spread of *Salmonella* in nursery pigs and assessing associated risk factors would be beneficial in developing control measures to prevent the circulation and maintenance of *Salmonella* in the later phases of production.

Newly weaned pigs are susceptible to disease because at this stage pigs are subjected to multiple stresses [15,16] and their immune system is not yet fully mature [17]. The control of endemic diseases in swine production often focuses on preventing newly weaned susceptible pigs from becoming infected with disease from older animals and thus perpetuating the disease within the herd. All-in and all-out management of nurseries combined with thorough cleaning between cohorts of weaned pigs is a common approach to prevent disease from cycling in this vulnerable population.

There are several challenges regarding the monitoring of *Salmonella* in the nursery to determine if weaned pigs are becoming actively infected with *Salmonella*. For example, most infected pigs become asymptomatic carriers, so the absence of clinical signs is not a good indication that *Salmonella* is not present [1]. In addition, infected pigs do not always shed *Salmonella* continuously or shed large numbers of bacteria and therefore negative results from bacterial culture may be misleading [1]. In addition, the best results for detecting *Salmonella* from an asymptomatic carrier pig is to use at least 25 g of fecal material [18,19] which is practical if the study design utilizes a pen sample, but it is more difficult to obtain a sufficient fecal sample from a specific individual pig. Serological testing methods to assess antibody response to *Salmonella* infection have been shown to be more effective in identifying the population of intermittent shedders than traditional bacteriological methods [7] but antibody titres in newly weaned pigs might be difficult to interpret because they may reflect either recent exposure or lingering passive immunity. Interpretation is clearer if antibodies from two time periods can be compared in order to verify that antibody levels are rising during the nursery stage, indicating exposure to *Salmonella* and not the presence of colostrum-derived immunity.

The objectives of this study were: (i) to identify Ontario swine nurseries with active *Salmonella* infections using serological and bacteriological testing methods and (ii) to determine risk factors associated with nursery cohorts with an active *Salmonella* infection.

## 2. Materials and Methods

The research was approved by the Animal Care Committee of the University of Guelph, in accordance with the guidelines set forward by the Canadian Council of Animal Care (Animal Utilization Protocol #3531). 

### 2.1. Study Design and Sampling

Forty-four Ontario swine farms with nursery barns were included in this study which took place between 2014 and 2019. Farm selection, based on the producer’s willingness to participate in the study, was both purposive and convenient. Along with conventional farms, antibiotic-free systems (including both certified organic and “raised without antibiotics” farms) were purposively selected to help capture a wide spectrum of farming practices in Ontario. A second cohort of nursery pigs was studied on six of these 44 swine farms resulting in a total of 50 study cohorts. These six farms were selected for a second cohort because they had been the first farms included in the study and thus over one year had passed since the first cohort was investigated and the producers were willing to continue to participate. The study involved following a weaned cohort of pigs through the nursery stage of production and involved two farm visits per cohort with the first visit (V1) at weaning or soon after weaning and the second visit (V2) near the end of the nursery stage.

For each cohort, the nursery was visited (V1) within a few days after the pigs were moved into the facility. On six of the nursery farms, where a second cohort of pigs was studied, the initial visit (V1) was conducted at weaning, immediately prior to the pigs being transferred to the nursery barn. In each of the 50 cohorts of weaned pigs, 20 animals were selected, with an attempt to choose pigs representative of the rooms and/or pens in the nursery barn (for example, if the pigs in a cohort were housed in 10 pens, then 2 average-sized pigs per pen would be selected for the study). The choice of an average-sized pig was based on subjective observation. The 20 pigs in each cohort were ear-tagged to provide individual identification and blood samples were collected from either the jugular vein or suborbital sinus. The cohort was visited a second time (V2) within a few days of the pigs being moved from the nursery to a grower barn. At V2, blood samples were collected from the same 20 pigs. Blood samples were centrifuged for 20 min at 1500× *g* and the sera were separated and stored at −20 °C. Individual rectal swabs were also collected from pigs at V2 and stored at −20 °C.

### 2.2. Questionnaire

During the first farm visit, a questionnaire was administered to the farm owner or manager and information was recorded by the investigator. The cohort-level questionnaire focused on management and biosecurity practices. Farm management questions included; the type of farm (farrow-to-finish, farrow-to-feeder, wean-to-finish, nursery only), production flow (continuous flow of pigs into and out of the nursery or all-in and all-out by room, or by building, or by site), and antibiotic use (certified organic, “antibiotic-free” or conventional). Basic production data and farm details such as herd size, source of nursery pigs, weaning age, length of stay in the nursery barn, and stocking density were also collected. Biosecurity practice questions included; whether there was a hospital pen, controlled entry for human traffic, downtime for visitors, the disposal method of deadstock, and cleaning methods between batches.

### 2.3. Salmonella Antibody Detection

Serum samples were assessed for presence of antibodies to *Salmonella* serogroups B, C, D, and E (O-antigens 1, 3–7, 9, 10, and 12) using an indirect ELISA (pigtype^®^ *Salmonella* Ab kit, QIAGEN, Leipzig, Germany). The ELISA was performed as described by the manufacturer. A sample-to-positive ratio (S/P) value was determined using the optical density (OD) values using the following equation:

*S/P* = (OD_sample_ − OD_negative control_)/(OD_positive control_ − OD_negative control_) (1)

The *S/P* ratio at V1 and V2 was identified as the antibody titre level at V1 and V2 for each pig. Based on the manufacturing guidelines, samples with a *S/P* ratio of ≥0.3 were identified as *Salmonella* seropositive and <0.3 were classified as *Salmonella* seronegative.

### 2.4. Salmonella Isolation

Rectal swabs were added to 9 mL of tetrathionate broth (TTB) (Becton Dickinson™, Franklin Lakes, NJ, USA) and incubated at 37 °C for 18 to 24 h. Then, 100 μL of TTB culture was transferred to 9.9 mL of Rappaport–Vassiliadis broth (RVB, Becton Dickinson™, Franklin Lakes, NJ, USA) and incubated at 41 °C for 18 to 24 h. Lastly, a loopful (~20 µL) of the RVB was plated onto xylose-lysine-tergoitol 4 (XLT4, Remel Thermo Fisher Scientific™, Lenexa, KS, USA) agar plates and incubated at 37 °C for 18 to 24 h. Plates with one or more *Salmonella* colonies were identified as *Salmonella* positive.

### 2.5. Data Analysis

Data were entered into Microsoft Excel for Mac 2019 Version 16.25 (Microsoft, Redmond, WA, USA). After cleaning, the data were imported into Stata (Stata/SE 14.2 for Mac; StataCorp, College Station, TX, USA) for further data management and descriptive analysis.

### 2.6. Salmonella Antibody Response Patterns

To explore the antibody response (based on the *S/P* ratio) in nursery pigs and to account for the possible presence of passive antibody titres at weaning, 8 possible patterns were established based on the pig’s *Salmonella* seropositivity status at V1 and V2, along with the change in direction of antibody response and whether the *S/P* ratio was ≥0.3 or not.

Specifically, pigs were considered serologically positive if the antibody titres from the two visits showed an increase in antibody titre (or the same antibody titre in both tests) resulting in a seropositive status at V2. All other patterns were considered serologically negative including a pattern with an initial high titre and a second lower S/P ratio even if it was ≥0.3. The assumption was that the decrease in antibody response reflects lingering passive immunity and not evidence of *Salmonella* exposure in the nursery.

A Python (Python v3.0.1, Fredericksberg, VA, USA) script was developed and performed to identify pigs into patterns (1–8). These results were presented and analyzed graphically. These pig-level data were also aggregated to the cohort-level.

### 2.7. Identifying Active Salmonella Infection in Nursery Cohorts

A cohort of nursery pigs was considered to have active *Salmonella* infection if one of the 20 pigs sampled tested positive by direct (culture) or by indirect (serological, antibody response patterns indicative of active infection) methods.

### 2.8. Agreement between Bacteriological and Serological Tests

The extent of agreement between the bacteriological and serological detection methods was determined using Cohen’s kappa (k) statistic. The kappa statistic was interpreted as follows: <0.2 slight agreement; 0.2–0.4 fair agreement; 0.4–0.6 moderate agreement; 0.6–0.8; substantial agreement; and >0.8 almost perfect agreement.

McNemar’s χ^2^ allowed assessment of the difference between the positive proportion of the bacteriology and serology testing methods. If the McNemar’s χ^2^ test was non-significant (*p* > 0.05), this would mean the proportions do not differ. Meanwhile, a significant McNemar’s χ^2^ test (*p* < 0.05) would mean there is a disagreement between the two testing methods and assessment of *kappa* would not be beneficial.

### 2.9. Risk Factors Associated with a Nursery Cohort with an Active Salmonella Infection

To assess risk factors associated with nursery barns with an active *Salmonella* infection, a logistic regression model was used. The active *Salmonella* infection (yes/no) of nursery barns was used as the dependent variable, while the second cohort conducted on six farms (farm cohort visit) was used as a fixed effect. Farm management and biosecurity practices, as explanatory variables, were initially screened using descriptive statistics and evaluated for collinearity using Spearman correlation coefficients. Continuous variables were assessed for linear relationships with the outcome graphically (lowess) and categorized if the relationship was not linear or quadratic. Explanatory variables were then independently assessed with the dependent variable using univariable analysis. The initial inclusion of variables in the model was based on a liberal *p* < 0.1. Using stepwise elimination, the full model was manually built excluding variables lacking statistical significance. The likelihood ratio test was used to assess the statistical significance (*p* < 0.05) of variables before removing them from the full model. Additionally, prior to exclusions, variables were tested for confounding to ensure there was less than 20% change in coefficients in main effects in the model. Since all explanatory variables were removed from the model, no further testing for interactions or fit of the model was required.

## 3. Results

### 3.1. Characteristics of the Nurseries

The 50 cohorts in this study included 19 (38%) farms that did not use antibiotics (including both certified organic farms and farms producing pigs for a program called “raised without antibiotics”) and the remaining cohorts were raised on conventional farms that used antibiotics (Table 1). Of 50 cohorts, 24 (48%) cohorts were located on a farrow-to-finish farm, while 17 (34%) cohorts were located on off-site nurseries (Table 1). The remaining 5 (10%) and 4 (8%) cohorts were nurseries associated with farrow-to-feeder pig and wean-to-finish operations, respectively. The flow of pigs varied as follows: 1/50 (2%) of the cohorts operated as an all-in/all-out (AIAO) flow by site, 25/50 (50%) of the cohorts operated as AIAO flow by building, and 13/50 (26%) cohorts AI/AO by room, and the remaining 11/50 (22%) cohorts used continuous pig flow in the nursery (Table 1).

With regard to cohort size, the population of nursery pigs ranged from 120 to 6500 (median = 2125, mean = 2235). Forty-one of 43 (95%) cohorts sourced nursery pigs from within their own production system, with this information missing from 7 participating nurseries. On average, 837 pigs entered the nursery at the same time, based on data from 41 cohorts (median = 650, min = 15, max = 2800). Based on the producers’ survey responses, the average weaning age of pigs varied from 18 days to 38.5 days-old (median = 21, mean = 23.2). On average, pigs spent approximately 38.7 days in the barn (ranging from 16 to 55 days, median 41). At V1, individual pigs ranged in age from 17 days to 41.5 days (median = 22, mean = 24.5). While at V2, pigs ranged in age from 50 to 85 days old (median = 64, mean = 63.9).

### 3.2. Salmonella: Antibody Titres, Seropositivity, Shedding

*Salmonella* antibody titres (based on the *S/P* ratio), seropositivity, and bacteriological results for nursery pigs at V1 and V2 are presented in Table 2. Overall, lower *Salmonella* antibody titres and number of pigs classified as *Salmonella* seropositive are observed in nursery pigs from V1 to V2. At the pig-level, 14.6% of pigs at the end of the nursery were found to be *Salmonella*-positive based on culture of a rectal swab.

Direct and indirect *Salmonella* testing results on a cohort basis are presented in Table 3. At the cohort level, 30 of 46 (65%) nursery cohorts had one or more pigs positive for *Salmonella* based on bacterial culture at V2 (Table 3). In terms of seropositivity, from weaning to the end of the nursery, 35 of 50 (70%) cohorts had a decrease in the number of seropositive pigs. Of the remaining cohorts 11 of 50 (22%) had an increase in the number of seropositive pigs, while 4 of 50 (8%) cohorts had no change in the percent of seropositive pigs (Table 3).

### 3.3. Salmonella Antibody Response Patterns

**Figure 1:**
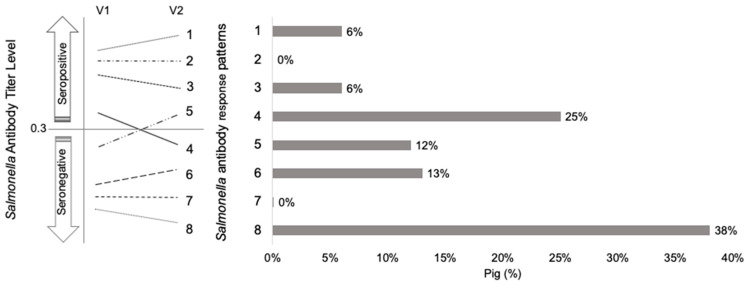
The change in *Salmonella* antibody titres in nursery pigs (*n* = 930, from 50 swine cohorts) from weaning, at visit 1 (V1), to the end of the nursery, at visit 2 (V2), is characterized into eight possible *Salmonella* antibody response patterns (1–8) ^a^. *Salmonella* seropositivity status (seropositive (Sp) if antibody titre level (based on *S/P* ratio) ≥0.3, seronegative (Sn) if antibody titre level <0.3).^a^ *Salmonella* antibody response patterns (1 to 8) 1—Sp at V1, increases in antibody titre 2—remains Sp (no change) 3—Sp at V1, decreases in antibody titre but remains Sp 4—Sp at V1, Sn at V2 5—Sn at V1, Sp at V2 6—Sn at V1, increases in antibody titre but remains Sn 7—remains Sn (no change) 8—Sn at V1, decrease in antibody titre.

At the individual level, 930 (69%) pigs had a decreasing *Salmonella* antibody response pattern (3, 4, 8) from V1 to V2 (Figure 1). Meanwhile, 31% of pigs were found to have an increase in antibody titre from V1 to V2 (patterns 1, 5, 6). No pigs were found to remain at baseline (patterns 2, 3). Based on antibody response patterns at the individual level, 18% of pigs were found to have an active *Salmonella* infection (patterns 1, 2, 5).

*Salmonella* antibody response patterns at the pig-level were aggregated to the cohort-level capturing the proportion of pigs following patterns 1 to 8 by cohort (Table 4). At the cohort level, 31/50 (62%) of nursery cohorts were identified as having an active *Salmonella* infection based on the serological criteria (Table 4).

### 3.4. Active Salmonella Infections

Although this study collected data from 50 Ontario nursery cohorts, complete bacteriological and serological testing information was only available on 46 nursery cohorts. A two-by-two table that identifies active *Salmonella* infections using bacteriological and serological detection methods is presented in Table 5. A non-significant McNemar’s χ^2^ indicated the positive proportions from the bacteriology and serological testing methods did not differ (McNemar’s χ^2^ = 0.07, *p =* 0.79). In addition, the k statistic revealed there was a fair agreement between the testing methods in identifying an active *Salmonella* infection on nursery cohorts (k = 0.29, *p* = 0.02).

Only 9 of 46 (20%) nursery cohorts were found to be negative for *Salmonella* using both testing methods. The remaining 37 of 46 (80%) nurseries were found positive either using serological or bacteriological methods or both. Specifically, 22 (48%) nursery cohorts were identified with an active *Salmonella* infection using both identification methods, while 8 (17%) and 7 (15%) were only positive based on serology alone and bacteriology alone, respectively.

### 3.5. Risk Factors Associated with Swine Cohort Having an Active Salmonella Infection

The association between risk factors and nursery cohorts with an active *Salmonella* infection was evaluated using a logistic regression model. With univariable analysis, an antibiotic-free system, the use of a shower, and the days the pigs spent in the nursery barn were found significant with a liberal *p*-value and included in the initial full model (*p* < 0.1). In the final model, no explanatory variables were significant or required in the model. Thus, no risk factors for active *Salmonella* infection in nursery barns were found.

## 4. Discussion

In this study, the epidemiology of *Salmonella* in nursery pigs was investigated on 44 farms (50 nursery cohorts) using both direct (culture) and indirect (serology) techniques to establish whether pigs were becoming infected during the nursery stage or not. Many pigs entered the nursery with high antibody titres to *Salmonella,* presumably reflecting circulating antibodies obtained via colostrum [16,20]. Overall, there was evidence that passive immunity declined during the nursery stage. The number of *Salmonella*-seropositive pigs was higher at the beginning of the nursery compared to the end of the nursery. The presence of this passive immunity, as pigs enter the nursery stage, provides some immune protection if challenged with *Salmonella* shortly after weaning [16], but the presence of passively-acquired antibodies makes interpretation of serological testing to determine active *Salmonella* infection in the nursery challenging. During the nursery, the passive immunity wanes and pigs begin to develop acquired immunity if pathogens are encountered [21], and therefore by using two testing points it is possible to identify pigs within a cohort that exhibit a serological pattern indicative of active infection. If pigs encounter *Salmonella* during the nursery barn after passive immunity disappears, it is likely for pigs to experience an increase in antibody titres [8,21]. But if pigs do not encounter *Salmonella* in the nursery barn, the decline in antibody titres continues. However, if piglets entering the nursery barn are *Salmonella* carriers, they are likely to shed *Salmonella* due to stress associated from weaning, change in environment, diet and comingling with different litters [4,8,15,17]. These animals and their pen-mates are likely to exhibit an increase in *Salmonella* antibody response.

Although there is a decline in *Salmonella* seropositivity from weaning to the end of the nursery in pigs, *Salmonella* is still present and circulating in many of these nursery cohorts. By understanding the *Salmonella* status at the cohort level, it can help veterinarians and producers better target the control and prevention methods in the nursery barn. Previous studies have used different methods and classification schemes for identifying *Salmonella* status in pig herds or scoring them as low, moderate and high-risk, using serological (based on different OD values/S/P ratios) and/or bacteriological testing methods [7,22,23,24,25]. Various studies have solely relied on either bacteriology or serology to identify *Salmonella* status in pigs and on farms [11,25,26]. To improve sensitivity, the present study used both bacteriological and serological detection methods to identify active *Salmonella* infections on nursery cohorts. Using the specified S/P ratio cut-off provided by the ELISA kit manufacturers, a nursery cohort was identified serological *Salmonella* positive (active infection) given 1 or more pigs were positive for *Salmonella* antibody response patterns 1, 2, or 5. The bacteriological identification of a nursery cohort as positive (active infection), based on if 1 or more pigs were shedding *Salmonella*, was in line with previous studies [7,23].

Traditionally, seropositivity in pigs is assessed at one time and is subsequently tested to monitor *Salmonella* status at the farm level. However, by using the *Salmonella* antibody response patterns approach introduced in the present study, the change in antibody response for each individual pig on a farm is identified into categories. This provides more detailed information on the *Salmonella* antibody response during the nursery stage (i.e., how many pigs are seroconverting on a farm, or how many pigs are increasing in antibody titres but remaining seropositive vs. seronegative, how many pigs are decreasing in antibody titres and becoming seronegative or remaining seropositive). In addition, since this method explores seropositivity status at weaning and at the end of the nursery along with the change in direction of antibody response, it decreases the likelihood of misinterpretation of passive immunity as a *Salmonella* infection in nursery pigs. Although there are strengths to this method, limitations include its inability to account for the magnitude of change, the initial antibody titre baseline and age at weaning. For example, pigs with an increase in antibody titres from 0.3 to 0.4 and 1.5 to 3.0 were identified as *Salmonella* antibody response pattern 1. By categorizing these pigs into the same pattern, data is lost. By incorporating the degree of change along with the initial antibody titre baseline into the *Salmonella* antibody response patterns, there can be better identification of the severity of *Salmonella* in nursery cohorts. The wide range in age at weaning presents a limitation as animals that were weaned at an older age may have improved *Salmonella* antibody response compared to animals that were weaned at a younger age.

In the present study, pigs following pattern 6 also had an increase in antibody titre but did not surpass the *S/P* ratio cut-off to be identified as seropositive. Kranker et al. [4] found a 60-day delay in the peak of seroprevalence after the peak in *Salmonella* culture prevalence. Surveillance of nursery cohorts with a large number of pigs following pattern 6 is important as it is possible that pigs following this pattern may become seropositive a few weeks after the second sample was taken, or in other words, they were exposed but were falsely classified as negative because of the delay in immune response. On the other hand, pigs following patterns (3, 4, and 8) where titres decrease during the nursery are likely experiencing a decrease in passive immunity and no indication that they are beginning to produce active immunity. However, because of the delay from exposure to antibody response, there is still a possibility of a false negative classification as well. Nursery pigs following pattern 4 and 8 are seronegative at the end of the nursery and would have been classified as negative based on a single serological sample at the end of the nursery. However, pigs following pattern 3 demonstrate a decrease in antibody titre but remain seropositive at the end of the nursery and based on a single test at the end of the nursery would generally be classified as positive. However, our interpretation of pigs showing this pattern is that the results reflect passive immunity and these pigs are categorized as *Salmonella* negative.

Using this technique of two sampling time points and identifying serological patterns coupled with direct culture of rectal swabs, a large portion of nurseries were identified as having an active *Salmonella* infection. Some nursery cohorts were identified as having an active infection using serology but would have been considered negative based on culture. Similarly, a few cohorts would have been considered negative by serology, but culture showed that *Salmonella* was indeed present. Although research has drawn correlation between bacteriological and serological classification, both these testing methods for *Salmonella* have strengths and weaknesses [7]. Results are somewhat dependent on timing of the test relative to the exposure to *Salmonella*. Serological testing relies on detecting antibodies and a positive response requires a few weeks to develop, whereas *Salmonella* culture is more likely to provide positive results shortly after exposure and becomes less reliable after a few weeks [4,7]. In general, bacteriological testing identifies a current infection whereas serological testing identifies historical exposure [7,27,28]. However, an advantage of serological testing is that it allows for the detection of intermittent shedders [4] and is a more inexpensive method with possibly better sensitivity in comparison to bacteriological testing [24].

In the present study, there is evidence of the shortcomings of relying solely on culturing rectal swabs to identify herds with active *Salmonella* infection because there were herds deemed positive using serology that were negative using culture. This might have been partly due to the use of rectal swabs instead of using a fecal sample of greater than 25 g, which has been advocated for monitoring purposes in populations of animals not exhibiting clinical signs of disease [18]. Likewise, there were herds considered positive based on culture but were considered negative using only serology. The specific ELISA used for this study detected antibody for *Salmonella* serogroups B, C, D, and E (O-antigens 1, 3, 4, 5,6, 7,9, 10, and 12), commonly found in Ontario. Serotyping *Salmonella* isolates, found in the present study, would have been beneficial to capture *Salmonella* serogroups that may have been unidentified by ELISA testing. This presents a limitation, impacting the serology results, because there is a possibility that some *Salmonella* infections went undetected. In addition, false negatives are a possibility due to laboratory errors during bacteriology and serological testing. This reinforces the argument for using the two testing methods in combination in order to more accurately identify positive herds.

The active *Salmonella* infection identified in 37 of 46 Ontario nursery cohorts (with complete information on both testing methods) strengthens the point that *Salmonella* is commonly present on Ontario pig farms, but importantly emphasizes that the nursery stage is a time in production when pigs often become infected and can then carry and spread *Salmonella* to the grower-finisher barns and eventually to the abattoirs. This information is useful for the timing and implementing of control and prevention strategies. For example, if farmers know that pigs are becoming exposed to *Salmonella* in the early nursery stage, then the vaccination of sows [29] or young pigs [30] might be implemented. Although a large portion of swine cohorts in this study were identified as having an active *Salmonella* infection, nine cohorts appeared to be *Salmonella*-free at the end of the nursery period, based on both serological and bacteriological methods and using a sample population of 20 pigs to represent the cohort. These results suggest that it is possible to have a negative population of pigs to send to the grower-finisher barn.

Examination of risk factors (e.g., AIAO by room, AIAO by barn, continuous flow, use of disinfection etc.) didn’t identify why the nine negative farms were different from nurseries with an active *Salmonella* infection. It is possibly that no associations were found due to the low variability in risk factors amongst nurseries with an active *Salmonella* infection compared to nurseries without an active *Salmonella* infection. Other studies attempting to identify risk factors associated with the presence of *Salmonella* have been inconsistent [9,14,26,31]. Most of these studies involved testing of pigs close to market weight or testing in abattoirs so their findings may not be relevant to nursery pigs. With the ability for *Salmonella* to survive long periods of time in the environment [32], it is likely that cleaning, disinfection, and biosecurity are important in reducing *Salmonella* in the nursery but the farm to farm variation in the implementation of these protocols are hard to capture in a survey and this aspect of *Salmonella* control requires further study. Future research should explore additional risk factors (i.e., feed management, antibiotic usage) and should include a greater number of nursery cohorts within depth reports on clinical sign related to salmonellosis.

## 5. Conclusions

Active *Salmonella* infection can be identified in a high proportion of swine nurseries. Using both serological and bacteriological testing methods in parallel improves the likelihood of identifying a nursery with active transmission of *Salmonella*. This study identified that both detection methods have strengths and weaknesses and by combining the techniques, researchers can better monitor active *Salmonella* infections on farms. Although the present study did not identify any risk factors, further work is warranted to investigate how cohorts of pigs in some nurseries remain negative for *Salmonella*.

## Figures and Tables

**Table 1:** Distribution of cohort-level risk factors (farm management and biosecurity) in 50 swine nursery cohorts (44 farms; 6 farms where a second cohort was conducted).

Variable	Level	N *	%
Production system—is this site a part of another production system	Yes	50	70
Production type	Farrow to finish	50	48
	Farrow to feeder		10
	Wean to finish		8
	Nursery (only)		34
Antibiotic-free system	Yes	50	38
Source of nursery pigs (within production)	Yes	43	95.3
Production flow	Continuous flow	50	22
	AIAO ** by room		26
	AIAO ** by building		50
	AIAO ** by site		2
Water	Fixed nipple	21	38.1
	Bowl drinker		28.6
	Nipple/bowl		28.6
	Swing nipple		4.8
Floor	Concrete/Solid	28	14.3
	Plastic Slates		85.7
Hospital pen	Yes	42	81
Shower	Yes	49	38.8
Danish entry	Yes	48	70.8
Dead stock disposal	Compost, burial or incineration within controlled access zone	42	31
	Compose, burial or incineration outside controlled access zone		21.4
	Third party pick up within		4.8
	Third party pick up outside		42.9
	Deliver to rendering		0
Cleaning between batches	Yes	42	97.6
Pre-soaking	Yes	41	70.7
Detergent	Yes	41	41.5
High pressure hot-water wash	Yes	41	75.6
Use disinfectant	Yes	41	90.2
Drying/down time	Yes	41	90.2
Salmonella vaccination of sows	Yes	43	0

* N values are applicable to the variable ** All-in/all-out (AIAO).

**Table 2:** *Salmonella* status (antibody titres, seropositivity, shedding) at visit 1 (V1; weaning) and visit 2 (V2; end of nursery) in nursery pigs in 50 cohorts (44 farms; 6 farms with a second cohort conducted).

	Visit	Mean	Std. Error	[95% CI]
Antibody titres ^a^	V1 (*n* = 966)	0.331	0.014	0.302, 0.359
	V2 (*n* = 961)	0.225	0.011	0.196, 0.253
	**Visit**	**Proportion (%)**	**Std. Error**	**[95% CI]**
Seropositivity ^b^	V1 (*n* = 966)	36.2	0.015	0.332, 0.393
	V2 (*n* = 961)	23.2	0.013	0.206, 0.260
Shedding	V2 (*n* = 830)	14.6	0.012	0.123, 0.172

^a^ Antibody titre values based on *S/P* ratio ^b^ seropositivity based on *S/P* ratio ≥0.3.

**Table 3:** Proportion of pigs that were *Salmonella* culture positive and seropositive (identified using enzyme-linked immunosorbent assay (ELISA) testing, seropositivity based on S/P ratio ≥0.3) at weaning (V1) and at the end of the nursery stage (V2). Data were collected from 50 swine cohorts (44 farms; 6 farms with a second cohort included in the study).

^ 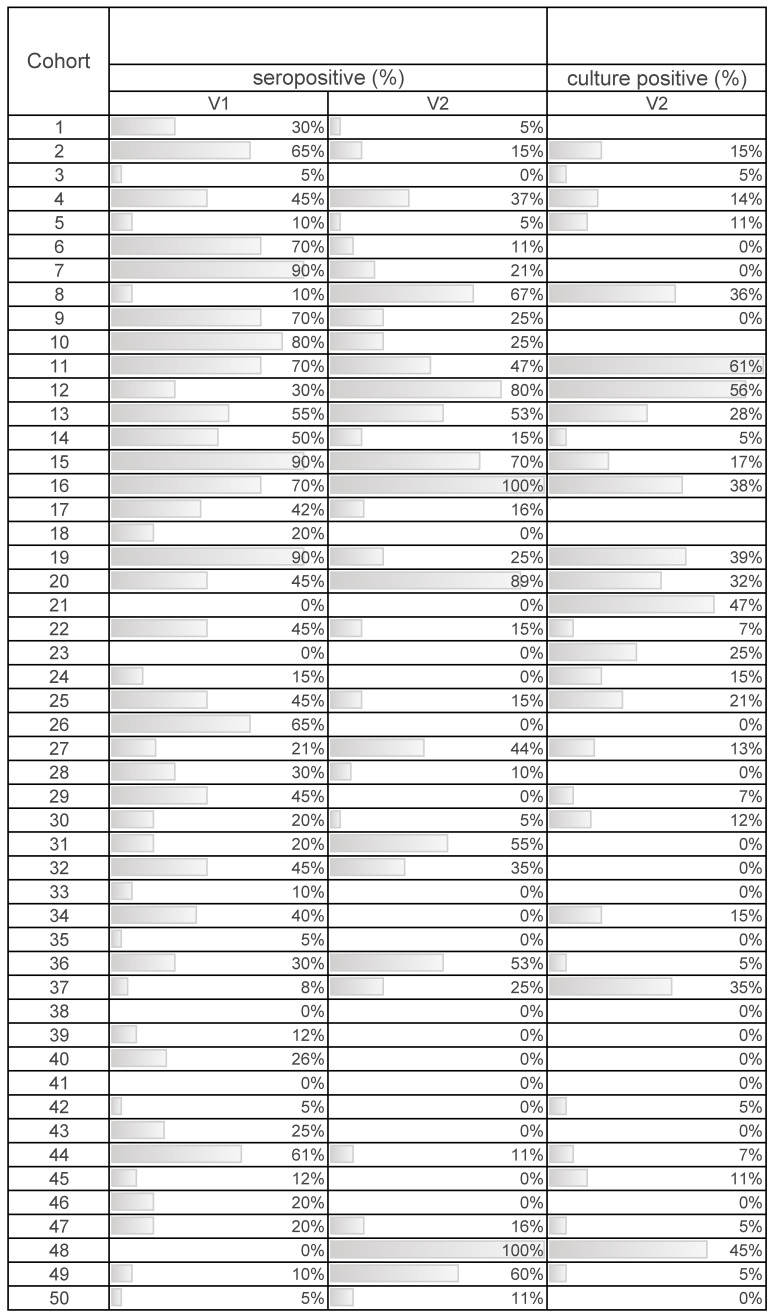 ^

**Table 4:** The change in *Salmonella* antibody titres in nursery pigs (*n* = 930) from visit 1 (V1; weaning) to visit 2 (V2; end of nursery) was characterized into eight *Salmonella* antibody response patterns (1 to 8) ^a^ and aggregated to the cohort level (*n* = 50) ^b^. This table captures the proportion of pigs following patterns 1 to 8 within each cohort.

^ 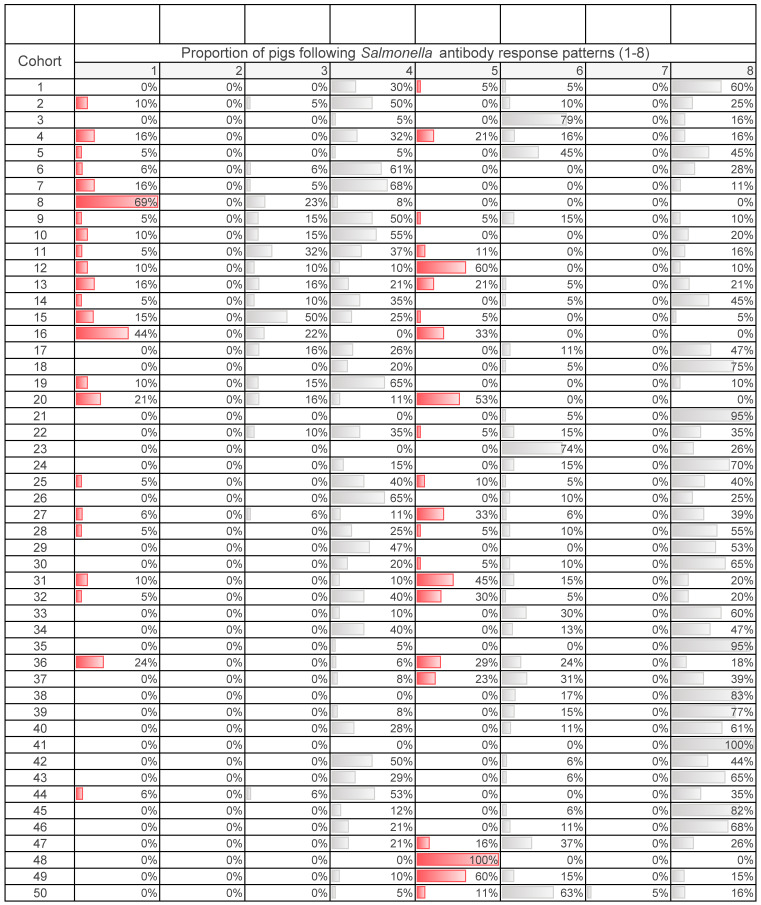 ^

^a^*Salmonella* antibody response patterns (1 to 8) 1—Sp at V1, increases in antibody titre 2—remains Sp (no change) 3—Sp at V1, decreases in antibody titre but remains Sp 4—Sp at V1, Sn at V2 5—Sn at V1, Sp at V2 6—Sn at V1, increases in antibody titre but remains Sn 7—remains Sn (no change) 8—Sn at V1, decrease in antibody titre ^b^ Data were collected from 50 swine cohorts (44 farms; 6 farms with a second cohort conducted) located in southwestern Ontario between 2014 to 2019. Red bars are used to identify pigs following *Salmonella* antibody response patterns (1, 2, 5) defined as having an active *Salmonella* infection.

**Table 5:** Active *Salmonella* infection on 46 Ontario nursery cohorts ^a^ identified using bacteriological (at the end of nursery (V2)) and serological (based on pattern of antibody response from weaning (V1) to the end of the nursery) testing methods.

		Active *Salmonella* Infection in Cohorts of Nursery Pigs		
		**Serology ^b^**		
		**+**	**-**	**Total**
**Bacteriology ^c^**	**+**	22 (48%)	8 (17%)	30
	**-**	7 (15%)	9 (20%)	16
	**Total**	29	17	46

^a^ Data were collected from 50 swine cohorts (44 farms; 6 farms with a second cohort studied). Complete bacteriological and serological testing information was only available from 46 nursery cohorts. ^b^ Serology positive: one or more pigs following *Salmonella* antibody response patterns with either no decrease or having an increase in *Salmonella* antibody titres from V1 to V2 resulting in *Salmonella* seropositivity at V2. ^c^ Bacteriology positive: one or more pigs shedding *Salmonella* in feces at V2.
